# Role of Voriconazole in the Management of Invasive Central Nervous System Aspergillosis: A Case Series from a Tertiary Care Centre in India

**DOI:** 10.3390/jof6030139

**Published:** 2020-08-18

**Authors:** Nitin Gupta, Parul Kodan, Ankit Mittal, Gagandeep Singh, George Netto, Prashant Ramteke, Sundeep Malla, Rohit Kumar, Tirlangi Praveen Kumar, Komal Singh, Anivita Aggarwal, Devashish Desai, Manish Soneja, Immaculata Xess, Naveet Wig

**Affiliations:** 1 Department of Medicine, All India Institute of Medical Sciences, New Delhi 110029, India; nityanitingupta@gmail.com (N.G.); parulkodan@yahoo.com (P.K.); mittalankit0505@gmail.com (A.M.); nettomundadan@gmail.com (G.N.); drrohitkgarg@gmail.com (R.K.); praveenkuma.124@gmail.com (T.P.K.); komalltmc@gmail.com (K.S.); anivita1513@gmail.com (A.A.); deva.desai17@gmail.com (D.D.); naveetwig@gmail.com (N.W.); 2 Department of Microbiology, All India Institute of Medical Sciences, New Delhi 110029, India; drgagandeep@gmail.com (G.S.); immaxess@gmail.com (I.X.); 3 Department of Pathology, All India Institute of Medical Sciences, New Delhi 110029, India; drprashantramteke@yahoo.com; 4 Department of Radiodiagnosis, All India Institute of Medical Sciences, New Delhi 110029, India; sundeepmalla1047@gmail.com

**Keywords:** sinocranial aspergillosis, cranial aspergilloma, septate hyphae

## Abstract

Invasive central nervous system (CNS) aspergillosis is acquired by either hematogenous dissemination or direct spread from a sinus infection. We describe a series of nine patients with CNS aspergillosis from a tertiary care teaching institute in North India who were treated with voriconazole alone or in combination with surgery. All patients who had clinical and radiological features consistent with fungal CNS infection, showed the presence of septate hyphae on histopathology/microscopy and were either culture positive for *Aspergillus* spp. or had serum galactomannan positivity were diagnosed as CNS aspergillosis. Clinical features, risk factors, diagnostic modalities, treatment details and outcome at last follow-up were recorded for all patients diagnosed with CNS aspergillosis. A total of nine patients were diagnosed with CNS aspergillosis. The median duration of presentation at our hospital was six months (IQR-2-9 months). Six patients had concomitant sinus involvement, while two patients had skull-base involvement as well. All patients were treated with voriconazole therapy, and three of these patients underwent surgery. All but one patient survived at the last follow-up (median duration was 14 months (IQR- 8-21.5). Two patients had complete resolution, and voriconazole was stopped at the last follow-up, and the rest of the patients were continued on voriconazole. Of the six patients who were continued on voriconazole, all but one had more than 50% radiological resolution on follow-up imaging. Invasive CNS aspergillosis is an important cause of CNS fungal infection that is often diagnosed late and requires long-term voriconazole-based therapy.

## 1. Introduction

Invasive aspergillosis involving the central nervous system (CNS) is a rare entity with high morbidity and mortality [1,2]. Most of the available literature on CNS aspergillosis describes it in immunosuppressed patients [1,2,3,4,5]. Decrease in the number (e.g., haematological malignancy, chemotherapy, transplant recipients) or function (e.g., chronic granulomatous disease, steroids) of neutrophils are considered as significant risk factors for invasive aspergillosis [3,4,5,6]. CNS aspergillosis is often diagnosed late due to the lack of awareness and non-specific presentation of the disease [1,2]. Delay in the diagnosis, angioinvasive nature of the fungi and poor penetration of antifungals in CNS makes it a challenging disease to manage. We describe a series of nine patients with CNS aspergillosis from a tertiary care teaching institute in North India who were treated with voriconazole-based therapy.

## 2. Materials and Methods

The patients were identified by manual review of the charts of all adult patients attending the Infectious disease clinic between July 2016 and July 2019 of All India Institute of Medical Sciences, New Delhi. All patients above the age of 14 years, with features consistent for CNS aspergillosis, were included in the study. Those patients where a minimum follow-up of three months was not available were not included in the study. The inpatient files of the identified patients were retrieved, and the patients were contacted for follow-up. Informed consent was obtained from the patients or next of kin (in case of deceased). All patients who had clinical and radiological features consistent with fungal CNS infection, showed the presence of septate hyphae on histopathology/microscopy, and were either culture positive for *Aspergillus* spp. or had serum galactomannan positivity were diagnosed as CNS aspergillosis. The cut-off optical density index value for serum galactomannan was taken as 0.7. Clinical and laboratory features of these patients were recorded using a case report form. The charts were also reviewed for the presence of established risk factors for aspergillosis. Involvement of sinuses, skull base and brain on computed axial tomography (CT) scan or magnetic resonance imaging (MRI) was also recorded with the help of a trained radiologist. The details of histopathology and culture of the biopsy were also recorded with the help of experienced pathologists and microbiologists. The medical (drug, dose, duration and therapeutic drug monitoring) and surgical treatment details were also recorded. The outcome at last follow-up was recorded as cured, died or ‘on-treatment’. Those patients who were ‘on-treatment’ were classified into >50% and <50% resolution based on the repeat imaging.

## 3. Results

**Figure 1:**
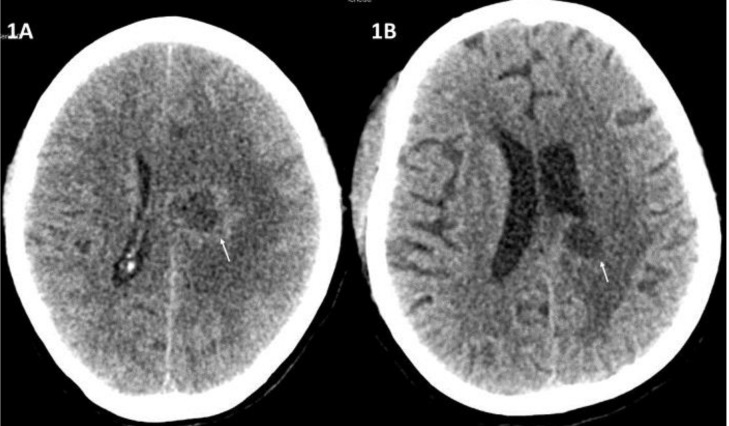
Contrast-enhanced computed tomography (CECT) axial sections of the brain showing a well-defined ring-enhancing lesion (arrow) in left centrum semiovale with perilesional oedema (**1A**). CECT brain post-treatment showing a significant decrease in the size of the lesion (arrow) as well as perilesional oedema (**1B**).

**Figure 2:**
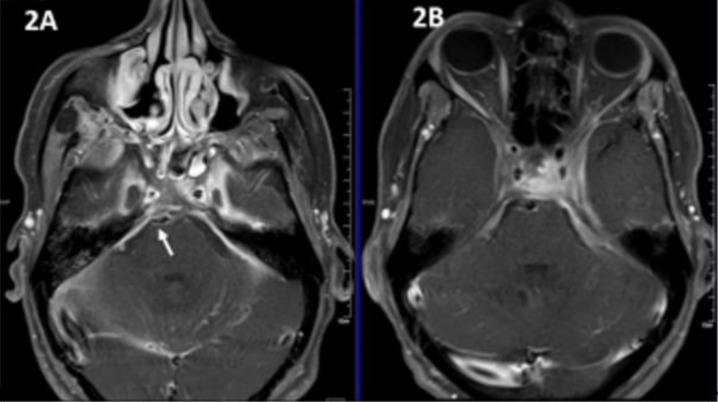
Post-contrast T1 weighted fat-saturated magnetic resonance imaging (MRI) images showing florid sinusitis with contiguous abnormal enhancement noted in bilateral cavernous sinuses and dura (arrow) (**2A**). Post-treatment MRI showing resolution of sinus inflammation with residual soft tissue in the left cavernous sinus and associated dural enhancement (**2B**).

**Figure 3:**
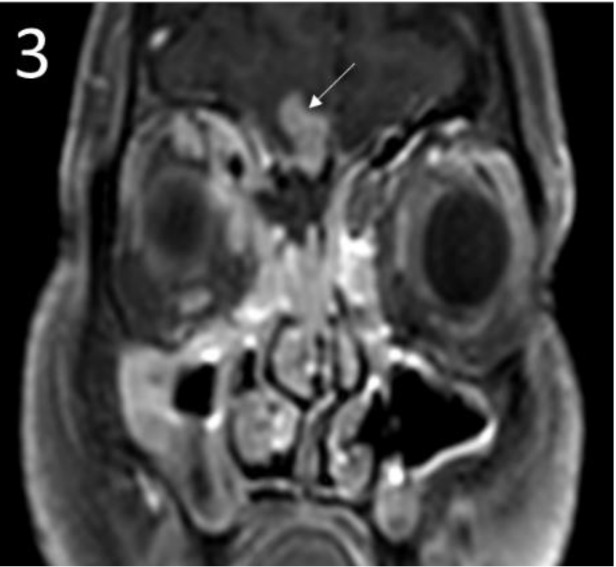
Post-contrast coronal images showing soft tissue enhancement in the right maxillary sinus, ethmoid sinuses with bony erosion and extension into right orbit and cranium (arrow).

**Figure 4:**
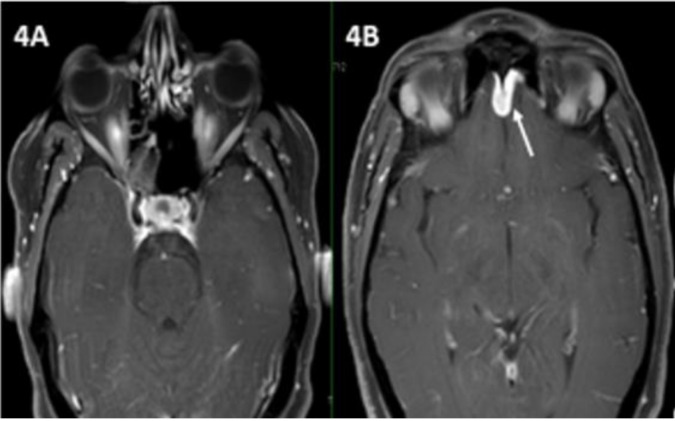
Post-contrast fat-saturated T1 weighted axial images showing sinusitis with abnormal nodular dural enhancement (arrow) in bilateral frontal location (**4A**,**4B**).

**Figure 5:**
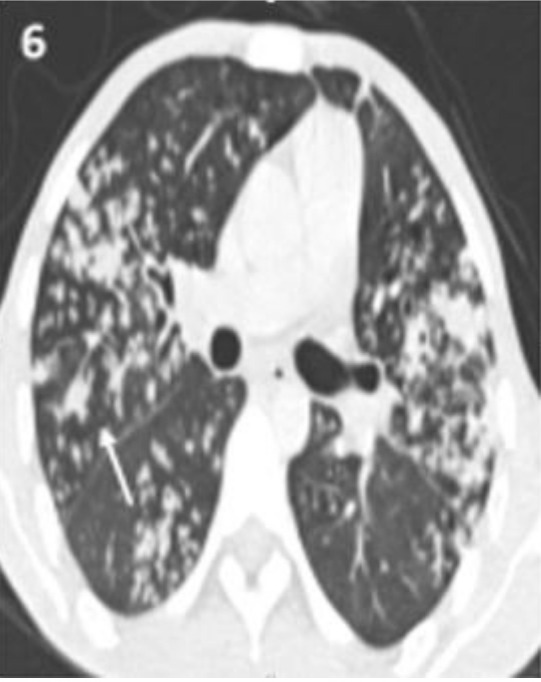
Ill-defined enhancing soft tissue centred in the left posterior ethmoidal air cells (asterisk) eroding the left sphenoid bone with extension into the left cavernous sinus and left middle cranial fossa (**5A**,**5B**).

**Figure 6:**
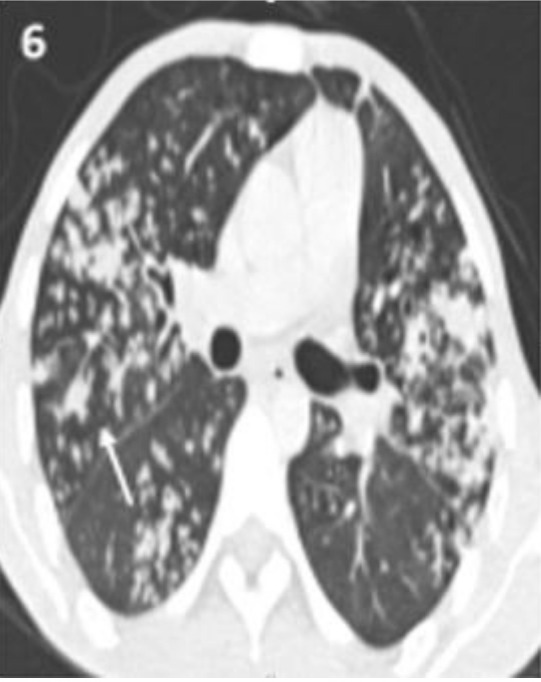
Lung involvement in a patient with brain abscess showing bilateral confluent/coalescing nodules (arrow) in airway distribution with no effusion or enlarged mediastinal nodes.

A total of nine patients were diagnosed with CNS aspergillosis between July 2016 and June 2019. Most of the patients were male (*n* = 7), and the mean (standard deviation) age was 37.9 (12.5) years. The age of the patients ranged from 19 to 55 years (Table 1). All nine patients were categorised as proven invasive fungal infection according to the 2019 update of the European Organization for Research and Treatment of Cancer and the Mycoses Study Group Education 7. Four patients had Type 2 Diabetes mellitus (DM), two patients were diagnosed with chronic granulomatous disease (CGD) on primary immunodeficiency workup, and one patient had a recent history of head injury. No risk factors were identified in three patients (Table 1). The median (interquartile) duration of symptoms at the time of presentation at our hospital was 6 [2,3,4,5,6,7,8,9] months. Six patients were diagnosed with sinocerebral aspergillosis, and two patients had concomitant skull-based osteomyelitis. The rest of the three patients had isolated brain abscess assumed to be of hematogenous origin. CT/MRI of the patients depicting isolated brain abscess and involvement of sinus with extension into cranial fossa/dura is compiled in Figure 1, Figure 2, Figure 3, Figure 4, Figure 5 and Figure 6. All the nine patients were diagnosed based on the presence of septate hyphae on histopathological examination. Of the six patients in whom fungal culture was sent, four were positive for *Aspergillus flavus*. Serum galactomannan was positive in only six out of the seven patients in whom the testing was done. In the patient, where serum galactomannan was negative, the galactomannan index in cerebrospinal fluid was more than 2. Extensive surgical debridement was done in three patients. All patients were treated initially with intravenous voriconazole therapy (6 mg/kg twice daily followed by 4 mg/kg twice daily). All the patients were shifted to oral voriconazole (200 mg twice daily) after discharge. Voriconazole was shifted to itraconazole after two months in one patient due to cost constraints. The dose of voriconazole was adjusted based on therapeutic drug monitoring in all the patients. Mortality was reported in only one patient who died despite three months of voriconazole therapy. The median duration of last-follow up was 14 months (IQR- 8-21.5 months). Two patients had complete resolution, and voriconazole was stopped at the last follow-up, and the rest of the patients were continued on voriconazole. No toxicities or issues with tolerability was reported with voriconazole in any of the patients. Of the six patients who were continued on voriconazole, all but one had >50% radiological resolution on follow-up imaging.

## 4. Discussion

In this series of nine cases of CNS aspergillosis, most of the patients were young and middle-aged adults. A review from India described young males working in an agricultural setting as the typical host for sinocerebral aspergillosis as their injured nasal mucosa are prone to getting colonised with the conidia in the air [8]. This may explain why most of our patients were young, with a mean age of 38 years. Similar to our series, other reports from India reported male preponderance and the mean age of presentation was 35–42 years [4,6,9]. Reports from developed countries showed a higher mean age of presentation (48–56 years) [2,10,11,12]. Most of the patients who presented to us lived in the neighbouring states and were referred to us only after the primary physicians were not able to make a diagnosis. This was probably the reason for delayed presentation. In another series from India, the mean duration of the presentation was 9.5 months [4]. A review of previous studies on CNS aspergillosis has been summarised in Table 2 [1,2,3,4,5,6,9,10,11,12,13,14].

Two distinct patterns of CNS involvement were noticed. The first pattern was isolated brain abscess, which was presumed to be of haematogenous origin, while the second pattern was the involvement of sinus followed by contiguous spread to the brain. In those patients with an isolated brain abscess, the primary site of involvement is considered to be the lungs (due to inhalation of spores) after which it gets disseminated through the blood to the brain [1,2,3,4,5,10,14]. In our series, three patients had isolated brain abscess, but concomitant lung involvement was noticed on chest radiography in only one patient. The pathogenesis of brain abscess formation is by obstruction of blood vessels by hyphae of *Aspergillus* spp. causing thrombosis, infarction and eventual evolution into an abscess [3]. *Aspergillus* spp. has an affinity for the distribution of perforating arteries. This is most likely due to the fungus affecting the origins of the perforating arteries [5]. Due to this affinity for the perforating arteries, the most commonly affected regions of the brain described in the literature are basal nuclei, corticomedullary junction and corpus callosum [5]. Similar to other series, the presenting complaints in our patients was a combination of fever, headache, altered sensorium and focal neurological deficits [1,2]. However, unlike other series, where it is described in predominantly immunosuppressed individuals (haematological malignancies, transplant recipients and patients on immunosuppressive drugs), our patients were apparently immunocompetent.

In those patients with sinus involvement, the CNS can be involved by a direct extension [2,3,4,5,6,9,11,13,14]. Concurrent skull base involvement is seen in a fraction of the patients with sinocranial aspergillosis [8,9,11,13]. In our series, six patients had sinus involvement, while two patients had concomitant skull-based osteomyelitis as well. Similar to our patients, most common presenting features are headache, nasal stuffiness, sinus fullness and visual loss. Compared to those affected by haematogenous dissemination, patients with sinus involvement are more commonly immunocompetent [14]. In our series, DM and CGD were found to be significant risk factors in some of these patients. Both these conditions may increase the risk of invasion by decreasing the phagocytic activity.

The radiological picture is often helpful in arousing suspicion of CNS fungal infection. In our patients with isolated brain abscess, ring-enhancing lesions were noted (Figure 1). The abscess in the brain showed an intermediate signal on T2-weighted MRI sequences in the centre and a hyperintense signal all around in the area involved. A similar pattern was described in other studies as well. In those with sinus involvement, there was inflammation of the involved paranasal sinuses with extension into surrounding structures. Similar to our patients, dural enhancement has been described as a prominent feature in rhinocerebral aspergillosis in some of the series (Figure 2 and Figure 3) [3]. A distinct nodular enhancement was noted in one of the cases (Figure 3). The involvement of brain parenchyma as an extension of sinus involvement was associated with ring-enhancing lesions (often multiple and irregular) in the frontal/temporal lobe. A similar pattern was described in other studies as well [3]. Involvement of other structures, such as orbits, skull base and cavernous sinus, was also seen.

Biopsy of the lesion and demonstration of septate hyphae is the most common modality for making a diagnosis of aspergillosis as the culture may be negative in some cases. Although cultures are helpful in speciation and tailoring the therapy, the specimens are often not sent to mycology laboratories due to a lack of adequate suspicion. In our series, the culture was sent for only six out of nine patients. Although *Aspergillus fumigatus* is the most common cause of invasive aspergillosis worldwide, *Aspergillus flavus* is more common in Asia and Africa [2,4,10,12,15]. This is presumably because of the improved survival of the spores of *A.flavus* in the tropical climate. Moreover, the larger size of the conidia of *A.flavus* results in them being trapped in the upper airways; therefore, it is more commonly associated with sinocranial aspergillosis [15]. Serum galactomannan is an important biomarker for invasive aspergillosis. Although it is reported to be less sensitive in non-neutropenic patients, in our series, it was positive in six out of seven patients [16]. It should also be noted that non-specific elevation of galactomannan has been described in the published literature [17].

In our series, all patients were treated with voriconazole-based therapy. Compared to amphotericin B and echinocandins that have poor CNS penetration, azole groups, in general, have good CNS penetration [18]. Amongst the azoles, fluconazole followed by voriconazole has the highest CNS penetration [19]. Considering the inadequate efficacy of fluconazole against *Aspergillus* spp., voriconazole remains the drug of choice for CNS aspergillosis. In a review of 123 cases of CNS aspergillosis, mortality with amphotericin monotherapy was higher when compared to voriconazole monotherapy (63 vs 35%) [10]. As voriconazole is prone to drug–drug interactions and some patients may be genetically poor metabolisers, wide variability in drug levels of voriconazole has been reported [20]. Moreover, toxicities associated with voriconazole use may be associated with poor compliance and the discontinuation of therapy [20]. To avoid this, the dosing of all the patients in our series was guided by therapeutic drug monitoring to maintain a trough level of 1-5μg/mL. This resulted in better efficacy and a decrease in adverse events. The use of voriconazole coupled with surgery (wherever possible) guided by therapeutic drug monitoring may have been the cause of the better outcome in our series compared to other series [1,2]. It is also worthwhile to note, similar to our series, the outcome of treatment in patients with primary sinus involvement is comparatively better than in those with hematogenous dissemination [10].

## 5. Conclusions

Our series demonstrated good outcome with the use of voriconazole guided by therapeutic drug monitoring in non-neutropenic individuals who were relatively young. The delayed diagnosis of these individuals highlights the need for early suspicion in patients with brain abscess with or without sinus involvement, especially with risk factors such as DM and CGD.

## Figures and Tables

**Table 1:** Summary of nine cases diagnosed with central nervous system (CNS) aspergillosis.

Pt Description		Clinical and Radiological Features	Duration of Symptoms (Months)	Microbiological/Pathological Features	Risk Factors	Antifungal tx	Surgery	Follow-Up			
Gender	R/U							Length (Months)	Tx	RI (%)	Alive
M	U	Headache, diplopia, ptosis, rhinosinusitis, orbital involvement, frontal lobe	6	Septate hyphae, Serum GM 1.3	CGD	AMP→VOR	No	25	VOR	>50	Yes
M	R	Facial pain, diplopia, rhinosinusitis, orbital involvement, pons	24	Septate hyphae, *Aspergillus* flavus on culture Serum GM not sent	None identified	VOR	No	21	VOR	>50	Yes
F	R	Headache, orbital involvement, rhinosinusitis, frontal lobe	6	Septate hyphae, Serum GM not sent, *Aspergillus flavus*	CGD	VOR	No	8	VOR	100	Yes
M	R	Headache, seizure, focal neurological deficits, brain abscess	2	Septate hyphae *Aspergillus flavus,* Serum GM-1	None identified	VOR	No	14	VOR	>50	Yes
M	U	Headache, focal neurological deficits, brain abscess	9	Septate hyphae, Serum GM 1.2 Culture negative	None identified	VOR	No	5	VOR	>50	Yes
M	U	Focal neurological deficits, rhinosinusitis, skull base osteomyelitis, temporal lobe	9	Septate hyphae, *Aspergillus flavus* on culture Serum 1.7	T2D (7.6%), Non-penetrating head trauma	VOR	Yes	8	VOR	>50	Yes
M	U	Facial pain, rhinosinusitis, skull base osteomyelitis, temporal lobe	8	Septate hyphae, Culture negative, serum GM 0.75	T2D (6.8%)	AMP→VOR	Yes	22	VOR	100	Yes
F	R	Fever, headache, focal neurological deficits, brain abscess	2	Septate hyphae, Serum GM 0.4 CSF GM 2	T2D (7.8%)	VOR	No	3	VOR	-	No
M	R	Fever, headache, vision loss, rhinosinusitis, orbital involvement, frontal lobe	2	Septate hyphae, Serum GM 0.7	T2D (6.9%)	VOR✕2 months → ITR	Yes	14	ITR	<50	Yes

Pt—Patient, R—rural, U—urban, M—Male, F—Female, T2D—type 2 diabetes with glycosylated haemoglobin in brackets, CGD—chronic granulomatous disease, GM—Galactomannan, ITR—itraconazole, AMP—amphotericin, VOR—voriconazole, tx—treatment, → followed by, d/c—discontinued, RI radiographic improvement.

**Table 2:** Review of retrospective studies (arranged country-wise and in order of year of study) reporting cases of CNS aspergillosis.

Study	Age (in years)	Sex (Male: Female)	Number of Patients	Year of Study	Number of Patients (Percentage)						
					Lung Involvement	Sinus Involvement	Skull Base Involvement	Risk Factors	Most Common *Aspergillus* spp.	Treatment	Mortality
Walsh et al. [1]	-	-	17	1956–1985	16 (94%)	1 (6%)	-	Immunosuppression-15 (88%)	-	Medical (Amphotericin)-2 (12%)	17 (100%)
Boes et al. [2]	Mean: 49 Range: 9–72	10:7	26	1981–1990	23 (88%)	3 (11%)	-	Immunosuppression-26 (100%)	*A.fumigatus*	Medical (Amphotericin)-23 (88%)	26 (100%)
Ashdown et al. [3]	Range: 5–87	7:4	11	1993	10 (91%)	1 (9%)	-	Immunosuppression-11 (100%)	-	Data not available	Data not available
DeLone et al. [5]			18	1998	Data not available	Data not available	-	Immunosuppression-18 (100%)	-	Data not available	16 (89%)
Kourkoumpetis et al. [10]	Mean: 56 Range: 31–71	8:6	14	2000–2011	11 (79%)	2 (14%)	-	Immunosuppression-14 (100%), History of traumatic brain injury-8 (57%)	*A.fumigatus*	Medical only-5 (36%)	5/5 (100%)
										Medical + Surgical-8 (57%)	2/8 (25%)
										No treatment-1 (7%)	1/1 (100%)
Murthy *et al.* [4]	Mean: 42 Range: 19–65	10:6	16	1990–1997	Data not available	16 (100%)	-	Agricultural/manual labourers-16 (100%)	*A.flavus*	Medical (Amphotericin +/- flucytosine) + Surgical-16 (100%)	6 (37%)
Saini et al. [6]	Mean: 35 Range: 9–55	6:6	12	2000–2010	Data not available	7 (58%)	-	DM-3 (25%)	-	Data not available	2 (17%)
Shah et al. [9]	Mean: 40 Range: 30–57	7:3	10	2016	0	10 (100%)	10/10 (100%)	Immunosuppression-0	-	Surgery + Medical therapy (voriconazole based)-10 (100%)	1 (10%)
Baeesa et al. [13]	Mean: 32 Range: 17–50	8:4	12	2000–2012	0	12 (100%)	10 (83.3%)	DM-4 (33%)	-	Medical (Amphotericin + azoles) + Surgical-12 (100%)	2 (17%)
Wang et al. [11]	Median: 48 Range: 22–64	4:4	8	2005–2015	0	4 (50%)	1 (12.5%)	Immunosuppression-2 (25%)	-	Medical only-5 (62.5%) Medical + Surgical-3 (37%)	5 (62.5%)
Marzolf et al. [14]			21	2006–2013	11 (52%)	8 (38%)	-	Immunosuppression-15 (71%)	-	Surgery-8 patients with sinus involvement	8 (38%)
Spapen et al. [12]	Mean: 50 Range: 44–68	4:6	10	2006–2011	9/10 (90%)	0	0	Immunosuppression 10 (100%)	*A.fumigatus*	Medical only (Voriconazole or amphotericin or both)-10 (100%)	9 (90%)
